# Development and clinical application of a endonuclease restriction real-time loop-mediated isothermal amplification (ERT-LAMP) assay for rapid detection of *Haemophilus influenzae*

**DOI:** 10.3389/fmicb.2022.1037343

**Published:** 2022-11-17

**Authors:** Jinzhi Cheng, Yuhong Zhou, Xue Zhao, Jingrun Lu, Jiahong Wu, Yu Wang

**Affiliations:** ^1^School of Basic Medical Sciences, Guizhou Medical University, Guiyang, China; ^2^School of Public Health, The Key Laboratory of Environmental Pollution Monitoring and Disease Control, Ministry of Education, Guizhou Medical University, Guiyang, China; ^3^Department of Clinical Laboratory, The First People’s Hospital of Guiyang, Guiyang, Guizhou, China

**Keywords:** *Haemophilus influenzae*, loop-mediated isothermal amplification, restriction endonuclease digestion, real-time fluorescence detection, *H. influenzae*-ERT-LAMP

## Abstract

*Haemophilus influenzae* is a main human pathogen that results in a series of diseases in children and adults, such as pneumonia, bacteremia, and meningitis. Although there are many detection methods, they cannot meet the requirements of an early diagnosis. For the prevention and control of *H. influenzae* infection, quick, sensitive, and particular diagnostics are crucial. Loop-mediated isothermal amplification (LAMP) coupled with restricted endonuclease digestion and real-time fluorescence (*H. influenzae*-ERT-LAMP) detection was employed to diagnose *H. influenzae*. *H. influenzae*-ERT-LAMP combines LAMP amplification, restriction endonuclease cleavage, and real-time fluorescence identification into a single-pot reaction, allowing for the rapid identification of *H. influenzae* in 40 min. The outer membrane protein (OMP) P6 gene of *H. influenzae* was employed to build a sequence of *H. influenzae*-ERT-LAMP primers. The limit of detection (LoD) of *H. influenzae*-ERT-LAMP test was 40 fg of genomic DNA per reaction, and the non-*H. influenzae* templates did not provide positive outcomes. To investigate the applicability of *H. influenzae*-ERT-LAMP method in clinical sample detection, 30 sputum specimens were obtained from individuals suspected of being infected with *H. influenzae*. *H. influenzae*-ERT-LAMP outcomes were in total agreement with LAMP-LFB and PCR. The *H. influenzae*-ERT-LAMP assay provides rapid, accurate, and sensitive detection making it a promising screening strategy in clinical and basic lab settings.

## Introduction

One of the primary pathogens of community-acquired respiratory airway infection in children is the gram-negative bacteria *Haemophilus influenzae*, which is often detected in the upper respiratory airways of healthy children and adults (Wang et al., 2008), predominantly leads to significant meningitis, epiglottitis, bacteremia, and pneumonia (Shah et al., 2021). However, the infection rate of *H. influenzae* type b (Hib) decreased after introducing the Hib vaccine, which reveals its effectiveness, but other non-Hib-causing invasive infections and antibiotic resistance are increasing (Wen et al., 2020). Therefore, for the early stage of *H. influenzae*, rapid and accurate identification is very necessary for disease prevention and control.

Fastidious in its requirements, *H. influenzae* needs NAD and an iron supply from hemoglobin, hematin, or hemin (Tristram et al., 2007). The morphology of *H. influenzae* colonies is difficult to differentiate from other spp. of *Haemophilus*, such as *Haemophilus parainfluenzae* (*H. parainfluenzae*). Moreover, traditional identification methods, such as population morphology, basic growth analysis, and serological determination, are also very time-consuming and complex, but they are still used in most laboratories (Tristram et al., 2007). As a result, cultivating and identifying in the clinic takes more time and effort. Rapid, sensitive, and specific methods must be established to obtain the fast and accurate identification of *H. influenzae*.

With the progression of molecular procedures, polymerase chain reaction (PCR) and PCR-based tests (e.g., single PCR, multiplex PCR, real-time PCR, and GeneXpert techniques) have been employed for the identification of *H. influenzae* (Van Ketel et al., 1990). Although these approaches have excellent analytical abilities, the necessities for special equipment, expensive reagents, and long testing procedures limit their application in nursing points and basic laboratories.

Loop-mediated isothermal amplification (LAMP) has been employed for the identification of *H. influenzae* in order to overcome the limitations of PCR technology (Kim et al., 2011; Diallo et al., 2021). LAMP requires four or two loop primers, which recognize six or eight or both regions on target profiles; nucleic acid amplification can be achieved with high efficiency using this method; it has been performed in recognition of many pathogens, like bacteria, viruses, fungi, and emerging/remerging infective agents (Notomi et al., 2000; Takano et al., 2019; Wang et al., 2021). Recently, there are several ways to identify LAMP amplification products, including using a specific fluorescent dye for dsDNA, electrophoresis of amplicons, turbidity due to magnesium pyrophosphate, nanoparticle-based lateral flow biosensor (LFB) and metal ion indicators (Wang et al., 2017). However, the judgment of outcome subjectivity, post-detection procedures require time, real-time detection cannot be achieved, and carryover contamination is a major problem with LAMP reactions (Iseki et al., 2007; Aonuma et al., 2010; Liang et al., 2012). Opening reaction tubes is unrecommended by a manufacturer of LAMP kits, or it requires separate equipment and facilities, further reducing the accuracy. This study introduces a novel endonuclease restriction real-time LAMP assay combined with real-time fluorescence detection. Then, the optimal reaction conditions, specificity, sensitivity and practicability of *H. influenzae*-ERT-LAMP assay were validated using strain pure cultures and clinical samples.

## Materials and methods

### Reagents and instruments

TianJin HuiDeXin Biotech Co., Ltd. (Tianjin, China) provided the DNA isothermal amplification kits, Nb.*BsrDI*, a polymer nanoparticle-based LFB, and visual detection reagent (VDR), while Baitaike Biotech Co., Ltd. provided the genomic DNA kit for nucleic acid sorting and purification (Beijing, China). Nano-Drop ND-2000 (Beijing, China) was employed to measure the purity and quantity of nucleic acid in A260/280.

### Design of *Haemophilus influenzae*-ERT-LAMP primers

**Figure 1:**
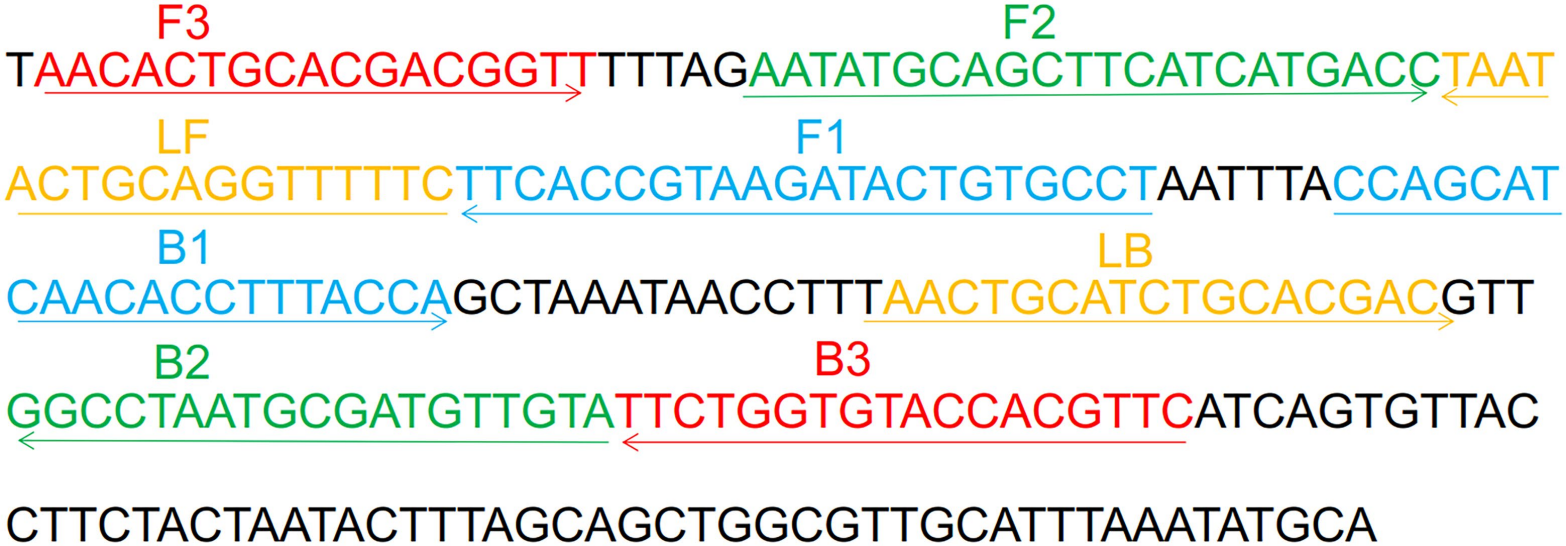
Sequence and location of OMP P6 gene used to design *Haemophilus influenzae*-ERT-LAMP primers. The nucleotide sequences of the sense strand of OMP P6 are listed. Right arrows and left arrows indicate sense and complementary sequences that are used.

A set of *H. influenzae*-ERT-LAMP primers was formed depending on the ERT-LAMP technology pathway using PrimerExplorer V5^1^ depending on the *H. influenzae* OMP P6 gene (Wang et al., 2016). A blast analysis showed that *H. influenzae*-ERT-LAMP primers are specific for *H. influenzae.* The dark quenchers employed were Black Hole Quencher-1, and the fluorophores utilized were FAM, which was monitored in real-time by the *H. influenzae*-ERT-LAMP system. The primers data (sequence, length, and alteration) is illustrated in Figure 1 and Table 1, and the length of the targeted sequence was 238 bp. All primers (HPLC purification level) were synthesized and purified by Tsingke Biotech Co., Ltd. (Kunming, China).

**Table 1:** Primers used in this study.

Assay type	Primers name^a^	Sequences and modifications (5′-3′)^b^	Length^c^	Gene
ERT-LAMP	F3	AACACTGCACGACGGTT	17 nt	OMPP6
	B3	GAACGTGGTACACCAGAA	18 nt	
	EFIP	5’-FAM-TGCAATGAGGCACAGT(BHQ1)ATCTTACGGTGAA-AATATGCAGCTTCATCATGACC-3’	51 mer	
	BIP	CCAGCATCAACACCTTTACCA-TACAACATCGCATTAGGCC	41 mer	
	LF	GAAAAACCTGCAGTATTA	18 nt	
	LB	AACTGCATCTGCACGAC	17 nt	
LAMP	F3	AACACTGCACGACGGTT	17 nt	
	B3	GAACGTGGTACACCAGAA	18 nt	
	FIP	AGGCACAGTATCTTACGGTGAA-AATATGCAGCTTCATCATGACC	45 mer	
	FIP*	biotin-AGGCACAGTATCTTACGGTGAA-AATATGCAGCTTCATCATGACC	45 mer	
	BIP	CCAGCATCAACACCTTTACCA-TACAACATCGCATTAGGCC	41 mer	
	LF	GAAAAACCTGCAGTATTA	18 nt	
	LF*	FAM-GAAAAACCTGCAGTATTA	18 nt	
	LB	AACTGCATCTGCACGAC	17 nt	
PCR	Hi-PCR-F	AACTTTTGGCGGTTACTCTG	20 nt	
	Hi-PCR-R	CTA ACACTGCACGACGGTTT	20 nt	

^a^FIP*, 5′-labeled with biotin when used in the LAMP-LFB assay; LF*, 5′-labeled with FAM when used in the LAMP-LFB assay; B, backward; BIP, backward inner primer; EFIP, new forward inner primer; F, forward; FIP, forward inner primer; LB, loop backward; LF, loop forward. ^b^FAM, 6-carboxy-fluorescein; BHQ1, black hole quencher 1. ^c^mer:monomeric; nt: nucleitide.

### Strains of bacteria and DNA preparation

This investigation included 20 clinically sorted strains, 3 *H. influenzae* strains, and 17 non-*H. influenzae* strains were employed, along with laboratory bacteria, reference strains (ATCC10211), and other strains (Table 2). All strains were employed to enrich and obtain genomic DNA templates (DNA minikits; Baitaike, Beijing, China). In order to utilize the obtained genomic templates, they were examined using an ultraviolet spectrophotometer (NanoDrop ND-2000, Beijing, China) at A260/280 and preserved at 20°C.

**Table 2:** The pathogen used in this study.

Bacteria species	Strain no. (source of strain)^a^	No. of strains	*Haemophilus influenzae-*ERT-LAMP^b^
*Haemophilus influenzae*	ATCC10211	1	P
*Haemophilus influenzae*	Isolated strains(GFPH)	3	P
*Salmonella*	ATCC14028	1	N
*Staphylococcus aureus*	ATCC29213	1	N
*Pseudomonas aeruginosa*	ATCC27853	1	N
*Candida albicans*	ATCC10231	1	N
*Escherichia coli*	ATCC25922	1	N
*Enterococcus faecalis*	ATCC29212	1	N
*Streptococcus pneumoniae*	ATCC49619	1	N
*Neisseria meningitidis*	ATCC13090	1	N
*Vibrio cholerae*	ATCC14731	1	N
*Staphylococcus epidermidis*	Isolated strains(GFPH)	1	N
*Enterococcus faecium*	Isolated strains(GFPH)	1	N
*Viridans streptococcus*	Isolated strains(GFPH)	1	N
*Proteusbacillus Vulgaris*	Isolated strains(GFPH)	1	N
*Acinetobacter baumannii*	Isolated strains(GFPH)	1	N
*Candida tropicalis*	Isolated strains(GFPH)	1	N
*Candida parapsilosis*	Isolated strains(GFPH)	1	N
*Stenotrophomonas maltophilia*	Isolated strains(GFPH)	1	N

^a^GFPH, The First People’s Hospital of Guiyang; ATCC, American Type Culture Collection. ^b^P, positive; N, negative. Only genomic DNA templates from *H. influenzae* could be detected by ERT-LAMP assay, indicating the extremely high specificity of the method.

### The standard *Haemophilus influenzae*-ERT-LAMP, LAMP-LFB and PCR reaction

To assess the feasibility of *H. influenzae*-ERT-LAMP primers, *H. influenzae*-ERT-LAMP amplification combinations were carried out in the last volume of 25 μl that included 0.4 μM EFIP primers, 0.4 μM BIP primers, 0.2 μM each LF and LB primers, 0.1 μM each F3 and B3 primers, 12.5 μl 2× reaction mix, 1 μl of *Bst* DNA polymerase, 1 μl of Nb.*BsrDI* endonuclease, 1 μl DNA model and double distilled water (ddH_2_O) were added to 25 μl. Applied Biosystems Co., Ltd.’s ABI 7500 real-time fluorescence PCR system, Eiken Chemical Co., Ltd.’s LA-500 real-time turbidity system, and agarose gel electrophoresis were utilized to evaluate the LAMP reactions and to optimize the amplification settings (such as the assay’s time and temperature). Utilizing ABI 7500 real-time system, the PCR parameters of holding phase at 65°C for 60 s, 40 rounds of denaturation at 65°C for 10 s, and extension at 65°C for 40 s were employed to observe the *H. influenzae*-ERT-LAMP combinations. FAM channels were employed to record fluorescence measurements concurrently.

The standard LAMP-LFB reaction was performed in a mixture of 25 μl (Wang et al., 2017). The reaction system was 25 μl and included a 2× reaction mix of 12.5 μl, 0.4 μM each FIP* and BIP primers, 0.2 M each of LF* and LB primers, 0.1 μM each of F3 and B3 primers, 1 μl (8 U) of *Bst* DNA polymerase, 1 μl of DNA template, and ddH_2_O were supplemented to 25 μl. In order to terminate the amplification response, it was incubated at 80°C for 5 min after 40 min at 65°C. LFB was employed to track all LAMP-LFB outcomes. In the LFB test, both CL and TL simultaneously emerged, indicating positive findings, while in the case of negative amplification, only CL was visible.

PCR amplification reaction combinations were performed in a 25 μl reaction volume, including 12.5 μl Premix Taq (TaKaRa), Hi-PCR-F primers 0.5 μl and Hi-PCR-R 0.5 μl, 1 μl of DNA template and ddH_2_O were added to 25 μl. To detect *H. influenzae*, the reaction environments were adapted to use 35 cycles, each relating 30 s denaturation at 94°C, 30 s of annealing at 50°C, and 30 s of addition at 72°C. Following the last cycle, all reactions were preserved for a further 10 min at 72°C. Our findings were displayed by resolution in agarose gel electrophoresis followed by ethidium bromide staining.

### Sensitivity and specificity of the *Haemophilus influenzae*-ERT-LAMP assay

To optimize the *H. influenzae-*ERT-LAMP test and examine the detection limit, 238 bp of the *H. influenzae* OMP P6 gene was chemically synthesized and cloned into pUC57 plasmid (herein referred to as pUC57-Hi-OMP P6 DNA) by Tsingke Biotech Co., Ltd. (Kunming, China), which contained the amplification target of *H. influenzae-*ERT-LAMP primers. The pUC57-Hi-OMP P6 DNA was used as a template for optimizing the H. influenzae-ERT-LAMP system and for the determination of sensitivity. The initial concentrations of pUC57-Hi-OMP P6 DNA were 4 μg, then ten-fold serial dilutions (400 pg, 40 pg, 4 pg, 400 fg, 40 fg, 4 fg, and 400 ag) of pUC57-Hi-OMP P6 DNA were arranged. The consecutive dilutions of pUC57-Hi-OMP P6 DNA were employed for identifying the limit of detection (LoD) of *H. influenzae-*ERT-LAMP, and a volume of 1 μl of these profiles were employed for *H. influenzae-*ERT-LAMP reactions.

For the *H. influenzae-*ERT-LAMP specificity examination, the *H. influenzae-*ERT-LAMP reaction was carried out in the environment illustrated above with pure genomic models from different strains (Table 2). Each specimen was examined separately at least twice.

### Applicability of the *Haemophilus influenzae*-ERT-LAMP assay to clinical samples

To evaluate the feasibility of *H. influenzae-*ERT-LAMP test in clinical specimen identification, we obtained 30 specimens of individuals who were suspected of infecting with *H. influenzae* at *the First People’s Hospital of Guiyang*. All sputum samples were confirmed as *H. influenzae* using LAMP-LFB and PCR. The gathering and examination of these DNA templates were authorized by *the First People’s Hospital of Guiyang* (Ethical approval No.G2020-S001).

## Results

### Confirmation of *H. influenzae*-ERT-LAMP products

**Figure 2:**
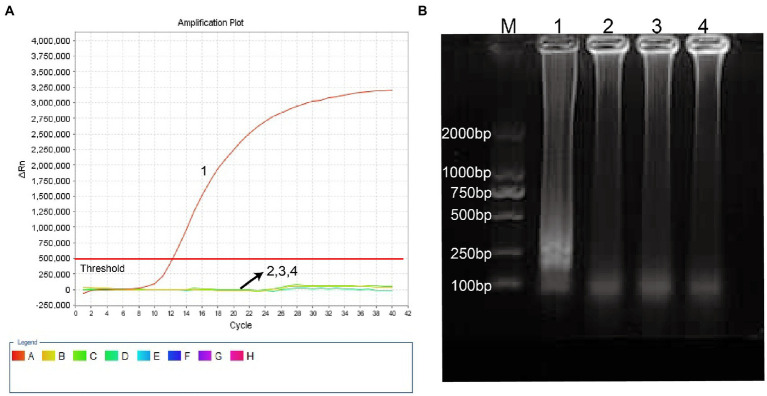
Identification and confirmation of *H. influenzae-*ERT-LAMP products. **(A)** the *H. influenzae-*ERT-LAMP was analyzed by means of real-time format, and the figures were obtained from FAM (labeling EFIP of P6 gene sequence) channels. Signals A1 indicate pUC57-Hi-OMP P6 DNA in FAM channels and signals A2, A3 and A4 indicate negative control (*Candida albicans*), negative control (*Escherichia coli*) and blank control (double-distilled water, DW). **(B)** agarose gel electrophoresis applied to *H. influenzae*-ERT-LAMP products; lane BM, DL 2000 bp DNA markers, lane B1 positive *H. influenzae*-ERT-LAMP products, lane B2, B3 and B4 indicate negative control (*Candida albicans*), negative control (*Escherichia coli*) and blank control (DW).

*Haemophilus influenzae*-ERT-LAMP method combines isothermal amplification, restriction endonuclease digestion, and real-time fluorescence detection in a single reaction vessel based on the standard (Iseki et al., 2007; Wang et al., 2016). Examine the reliability of the ERT-LAMP primers for the H. influenzae test (Table 1). Employing pUC57-Hi-OMP P6 DNA as a template, *H. influenzae*-ERT-LAMP combinations were run for 40 min at a fixed temperature of 65°C. Next, real-time fluorescence PCR and agarose gel electrophoresis were employed to verify amplification results. Figure 2A shows that the quenching production was detected utilizing a 7,500 real-time PCR system as a strong rise in FAM signals in the positive findings but not in the negative or blank controls. The *H. influenzae*-ERT-LAMP products were then electrophoresed to confirm that the anticipated ladder bands were present (Figure 2B).

Hence, the ERT-LAMP primers for OMP P6 gene recognition in the present research effectively established the *H. influenzae-*ERT-LAMP assay. Also, the best reaction temperature for *H. influenzae-*ERT-LAMP was established, with 67°C being the highest possible option for *H. influenzae-*ERT-LAMP reaction (Supplementary Figure S1).

### Evaluation of sensitivity of the *Haemophilus influenzae*-ERT-LAMP assay

**Figure 3:**
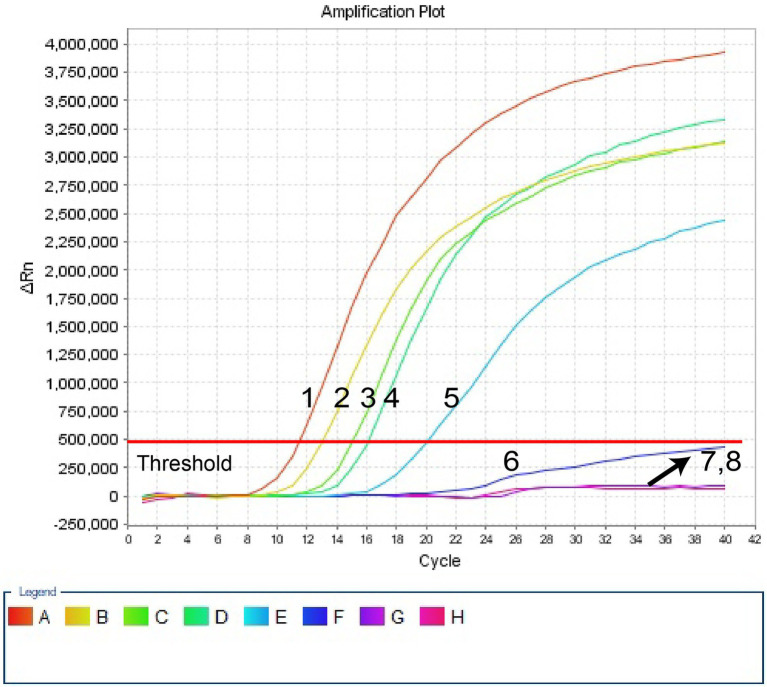
Sensitivity of *H. influenzae-*ERT-LAMP assay. Signals 1–7 indicate a series of dilutions (400 pg, 40 pg, 4 pg, 400 fg, 40 fg, 4 fg, 400 ag) of pUC57-Hi-OMP P6 DNA and a blank control (DW) were operated according to standard *H. influenzae-*ERT-LAMP reactions.

Ten-fold serial dilutions of pUC57-Hi-OMP P6 DNA, from 400 pg to 400 ag copies per 1 μl, were employed to detect the sensitivity of *H. influenzae-*ERT-LAMP test. Quenching production can be observed as a vigorous rise in FAM signals, and positive amplification can be noticed in about 40 min. The LoD of *H. influenzae-*ERT-LAMP test for identifying pUC57-Hi-OMP P6 DNA was 40 fg of genomic DNA per tube (Figure 3).

### Analytical specificity of the *Haemophilus influenzae*-ERT-LAMP assay

**Figure 4:**
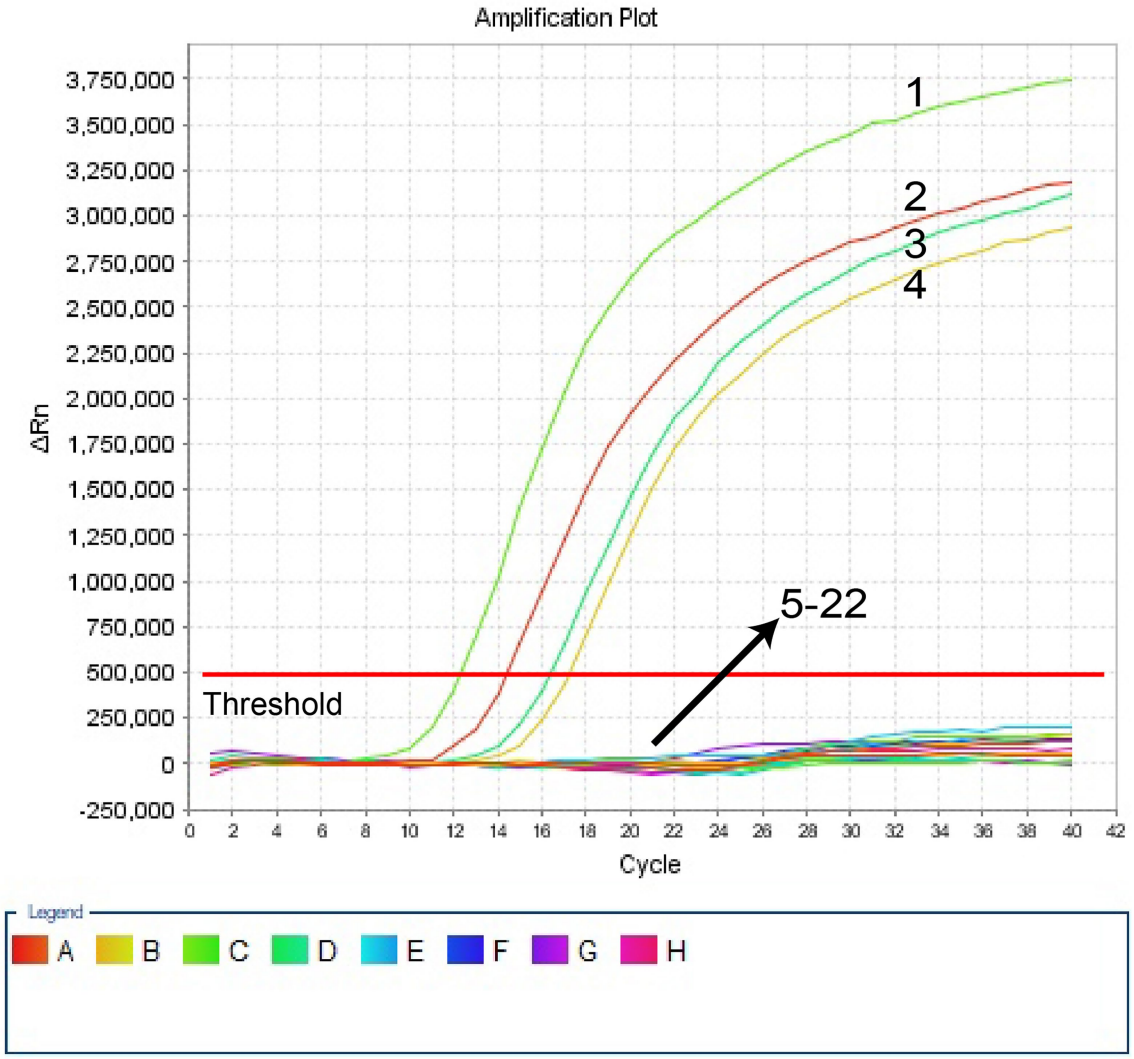
Specificity of *H. influenzae-*ERT-LAMP detection for different strains. The *H. influenzae*-ERT-LAMP amplifications were performed using different genomic DNA templates and were monitored by means of real-time detection. Signals 1, Positive control (*H. influenzae* ATCC10211), signals 2–4, *H. influenzae* isolated strains; signals 5, *Salmonella* ATCC14028; signals 6, *Staphylococcus aureus* ATCC29213 signals 7, *Pseudomonas aeruginosa* ATCC27853; signals 8, *Candida albicans* ATCC10231; signals 9, *Escherichia coli* ATCC25922; signals 10, *Enterococcus faecalis* ATCC29212; signals 11, *Streptococcus pneumoniae* ATCC49619; signals 12, *Neisseria meningitidis* ATCC13090; signals 13, *Vibrio cholerae* ATCC14731; signals 14, *Staphylococcus epidermidis* isolated strains; signals 15, *Enterococcus faecium* isolated strains; signals 16, *Viridans streptococcus* isolated strains; signals 17, *Proteusbacillus Vulgaris* isolated strains; signals 18, *Acinetobacter baumannii* isolated strains; signals 19, *Candida tropicalis* isolated strains; signals 20, *Candida parapsilosis* isolated strains; signals 21, *Stenotrophomonas maltophilia* isolated strains; signals 22, a blank control (DW).

In this investigation, the specificity of *H. influenzae-*ERT-LAMP test was assessed with the genomic templates obtained from the reference strains ATCC10211, 3 *H. infuenzae* strains, and 17 *non-H. infuenzae* bacterial pathogens (Table 2). Figure 4 reveals that the positive outcomes were particularly yielded with the genomic DNA from *H. infuenzae*, while the negative outcomes were identified with *non-H. infuenzae* strains. These outcomes revealed that the *H. influenzae-*ERT-LAMP test defined here was definite to target sequence detection.

### Evaluation of the *Haemophilus influenzae*-ERT-LAMP assay by using clinical sample

**Figure 5:**
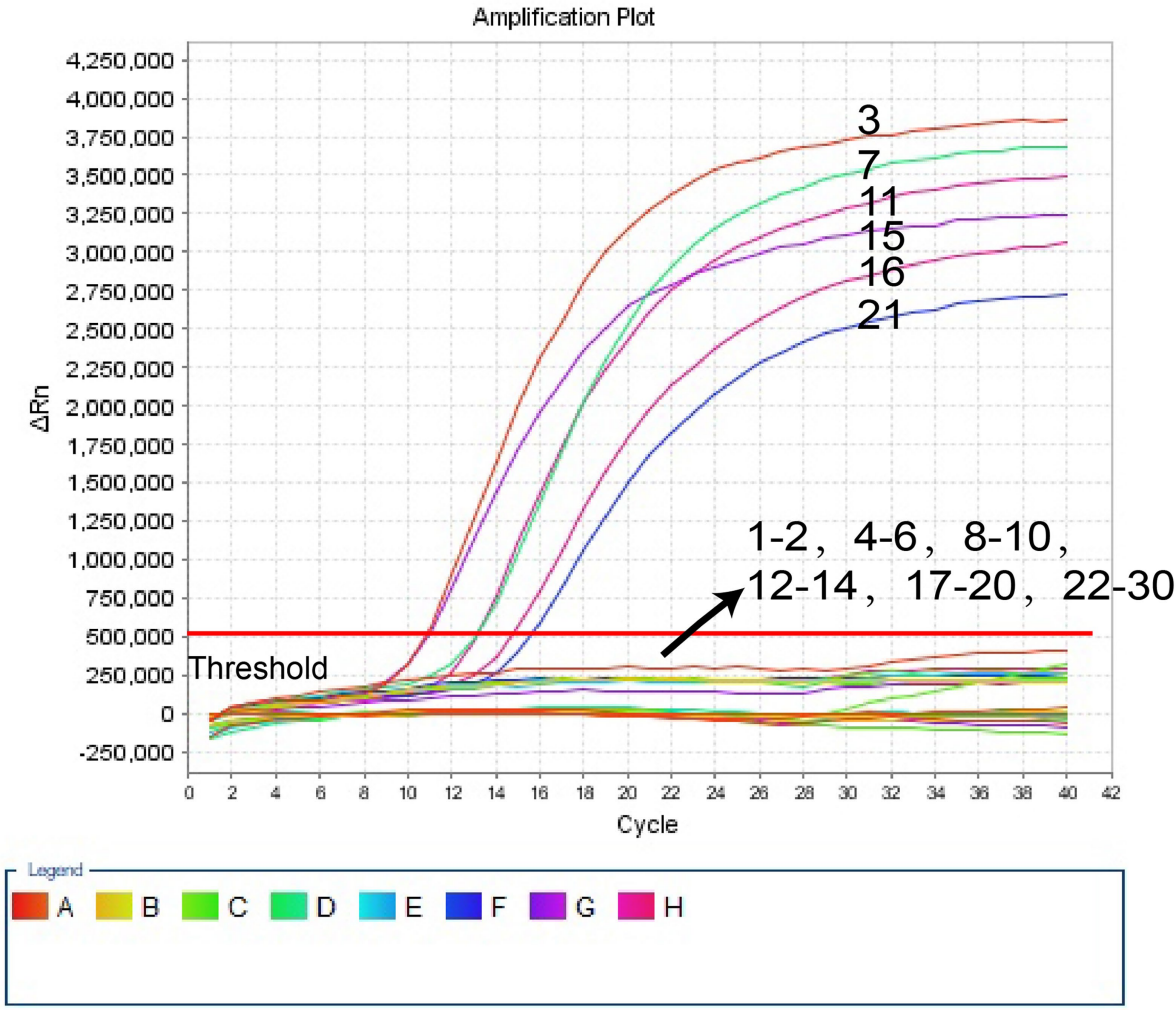
The *H. influenzae-*ERT-LAMP assay for detecting *H. influenzae* in clinical samples. Signals 3, 7, 11, 15, 16, 21 indicate *H. influenzae* in clinical samples in FAM channels. Signals 1–2, 4–6, 8–10, 12–14, 17–20, 22–30 represented the negative results.

**Figure 6:**
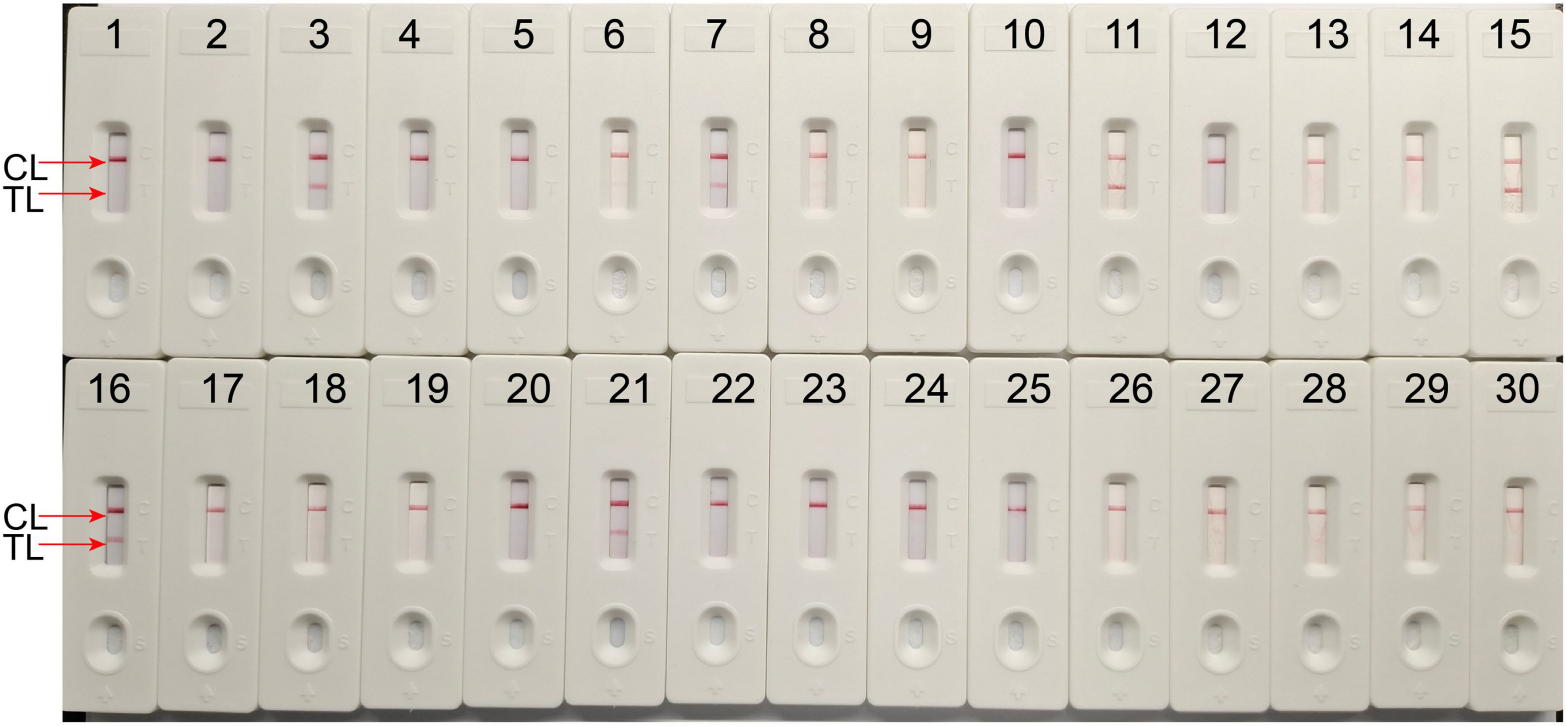
The LAMP-LFB assay for detecting *H. influenzae* in clinical samples. Later flow biosensor was applied for detecting LAMP amplicons. The numbers 3, 7, 11, 15, 16, 21 represented the positive results. 1–2, 4–6, 8–10, 12–14, 17–20, 22–30 represented the negative results.

**Figure 7:**
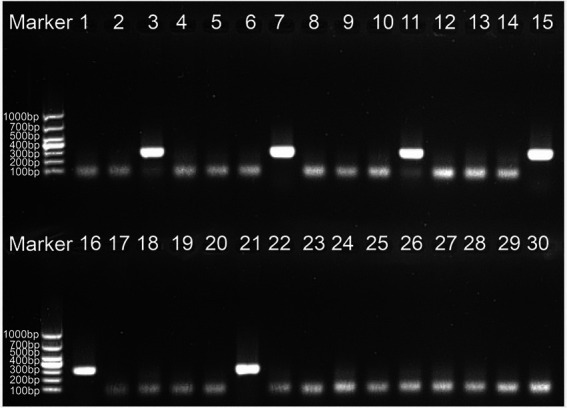
The PCR assay for detecting *H. influenzae* in clinical samples. The numbers 3, 7, 11, 15, 16, 21 represented the positive results. 1–2, 4–6, 8–10, 12–14, 17–20, 22–30 represented the negative results.

To define the applied application of novel *H. influenzae-*ERT-LAMP detection of *H. influenzae* in a clinical sample, 30 sputum samples were divided into three equal parts and then simultaneously detected using the *H. influenzae-*ERT-LAMP, LAMP-LFB assay, and PCR assays. Of the 30 sputum samples, 6 samples were *H. influenzae* positive (Table 3)*. H. influenzae-*ERT-LAMP results (Figure 5) showed completely in accordance with the LAMP-LFB (Figure 6) and PCR asssy (Figure 7). These outcomes demonstrated that the *H. influenzae*-ERT-LAMP assay offers a rapid, reliable, and sensitive recognition of *H. influenzae*, which can be employed as a possible screening method for *H. influenzae* in the clinical and basic laboratory environment.

**Table 3:** Comparison of *H. influenzae*-ERT-LAMP, LAMP-LFB, and PCR Assays for the Detection of *H. influenzae.*

Detection method^a^	Sputum samples (*n* = 30)		Time consumption (minutes)
	Positive	Negative	
*H. influenzae*-ERT-LAMP	6	24	40
LAMP-LFB	6	24	45
PCR	6	24	180

^a^*H. influenzae*-ERT-LAMP, *H. influenzae* endonuclease restriction real-time loop-mediated isothermal amplification.

## Discussion

The World Health Organization (WHO) closely monitors *H. influenzae*, which mostly impacts children under five and people over 65 years of age. It is alleged to be the second most prevalent bacterial infection causing pneumonia in Chinese children (Heliodoro et al., 2020). Hib vaccine is a beneficial and affordable intervention to protect children in mainland China against pneumonia, meningitis, and other illnesses that can be prevented by vaccination (Ning et al., 2018). Non-typeable *H. influenzae* (NTHi) strains have been a worldwide issue since the Hib vaccine was developed since they mostly induce the upper respiratory tract, otitis media, and severe invasive illness (Li et al., 2020). Therefore, it is crucial to distinguish *H. influenzae* quickly, accurately, and sensitively from other pathogenic respiratory tract organisms in order to stop and manage *H. influenzae* outbreaks. Traditional detection techniques, such as population morphology, basic growth analysis, and serological testing, often fall short of the time and sensitivity required for rapid detection.

Herein, we document the new *H. influenzae*-ERT-LAMP chemistry, which successfully combined the conventional LAMP chemistry and restriction endonuclease digestion with fluorescent reporter dye in real-time for checking the result of LAMP products during each amplification cycle, was developed and assessed for nucleic acid examination of clinical samples. Previous studies have demonstrated that *H. influenzae* OMP P6 gene, which is a highly conserved gene and has become a potential vaccine component and is more suitable for the identification of *H. influenzae* than other genes (Murphy et al., 1986; Nelson et al., 1991; Karalus and Murphy, 1999). Therefore, OMP P6 gene was chosen as the target gene for rapid diagnosis of *H. influenzae*.

In our study, *H. influenzae*-ERT-LAMP only needs a reasonably simple fluorescent tool to preserve a fixed temperature for 40 min (Figure 3). As compared to *H. influenzae* LAMP techniques used in previous studies, the *H. influenzae*-ERT-LAMP method detected *Haemophilus influenzae* results in real-time by real-time fluorescence examination, which excludes the requests of particular reagents (e.g., pH indicators), complicated processes (e.g., electrophoresis) and costly devices (e.g., real-time turbidity; Cao et al., 2022). Furthermore, carryover contamination is effectively avoided by closing the *H. influenzae*-ERT-LAMP reaction tubes during the experiment. However, due to the need for COVID-19 detection, PCR laboratories have been established in all hospitals above the second level of Chinese mainland, and *H. influenzae*-ERT-LAMP assay has a good prospect of popularization and application (Wang et al., 2021).

The whole procedure of *H. influenzae*-ERT-LAMP recognition, containing genomic DNA template development (20 min) and ERT-LAMP reaction (40 min), was completed within 60 min, improving the usage of a temperature-modulating device, and does not need more agarose gel identification or pyrosequencing. In addition, *H. influenzae*-ERT-LAMP has a high sensitivity. The LoD of *H. influenzae*-ERT-LAMP assay was 40 fg of each genomic DNA per reaction and 1–1,000 times more than that of the PCR method (Falla et al., 1994; Corless et al., 2001; Tian et al., 2012). It can amplify the targets at the LoD level in a shorter time, thus considerably reducing the total test time (Table 3).

In addition to high sensitivity, *H. influenzae*-ERT-LAMP test is highly specific. For *the H. influenzae-*ERT-LAMP assay specificity test, positive findings were reported for *H. influenzae* samples; however, non-*H. influenzae* strains did not produce any positive amplifications (Figure 4). Furthermore, the unique *H. influenzae*-ERT-LAMP test proposed here can accurately distinguish target sequences with elevated specificity, perform in a single isothermal amplification stage, and provide interpretable data (Wang et al., 2016). For a further detailed evaluation of the practicality of *H. influenzae*-ERT-LAMP method to target pathogens, 30 clinical sputum samples were randomly tested using *H. influenzae*-ERT-LAMP, LAMP-LFB detection, and PCR, respectively. *H. influenzae*-ERT-LAMP assay showed elevated specificity for *H. influenzae* strains in sputum samples, consistent with the LAMP-LFB and PCR method (Table 3). Based on these characteristics, *H. influenzae*-ERT-LAMP procedure is technically simple, quick, and low cost, delivering applied solutions for medical and disease control laboratories, particularly in reduced resource parameters.

## Conclusion

We concluded that *H. influenzae*-ERT-LAMP assay targeted the OMP P6 gene of *H. infuenzae* and was effectively designed in the present study. In the application and assessment procedure, *H. influenzae*-ERT-LAMP method showed good specificity and sensitivity by detecting reference strains and clinical samples. Therefore, the *H. influenzae*-ERT-LAMP assay provides a novel choice for reliable, quick and simple detection of *H. influenzae*.

## Data availability statement

The original contributions presented in the study are included in the article/Supplementary material, further inquiries can be directed to the corresponding author.

## Ethics statement

The investigation was authorized by the Human Ethics Committee of *the First People’s Hospital of Guiyang* (Approval No. G2020-S001) and acts following the Declaration of Helsinki. Prior to receiving the samples/isolates and performing the research, the monitoring stations erased all identifying information from the individuals suspected of being infected with *H. influenzae*. The Human Ethics Committee of t*he First People’s Hospital in Guiyang* waived the patient’s informed consent.

## Author contributions

JC, YZ, and YW conceived and designed the experiments. YZ, XZ, JC, and JL performed the experiments. YZ, YW, XZ, and JW analyzed the data. XZ, JC, and YW wrote the paper. All authors contributed to the article and approved the submitted version.

## Funding

This study was supported by Zhu ke he tong [2020]-10-6, [2019] Zhu wei jian ke ji he tong zi di 001 and Zhu ke he tong [2021]-43-25 from the Science and Technology Department of Guiyang city of Guizhou Province, and grant (Qian Ke He Zhi Cheng [2021] Yi Ban 440) from Science and Technology Department of Guizhou Province.

## Conflict of interest

The authors declare that the research was conducted in the absence of any commercial or financial relationships that could be construed as a potential conflict of interest.

## Publisher’s note

All claims expressed in this article are solely those of the authors and do not necessarily represent those of their affiliated organizations, or those of the publisher, the editors and the reviewers. Any product that may be evaluated in this article, or claim that may be made by its manufacturer, is not guaranteed or endorsed by the publisher.

## Supplementary material

The Supplementary material for this article can be found online at: https://www.frontiersin.org/articles/10.3389/fmicb.2022.1037343/full#supplementary-material

## Footnotes

^1^ https://primerexplorer.jp
