# Supplementary material for: Development and clinical application of a endonuclease restriction real-time loop-mediated isothermal amplification (ERT-LAMP) assay for rapid detection of Haemophilus influenzae
Source: Front Microbiol. 2022 Nov 17;13:1037343. doi: 10.3389/fmicb.2022.1037343 (PMC9712716; doi:10.3389/fmicb.2022.1037343)
Supplement: Supplementary file 1 [file Image_1.pdf]

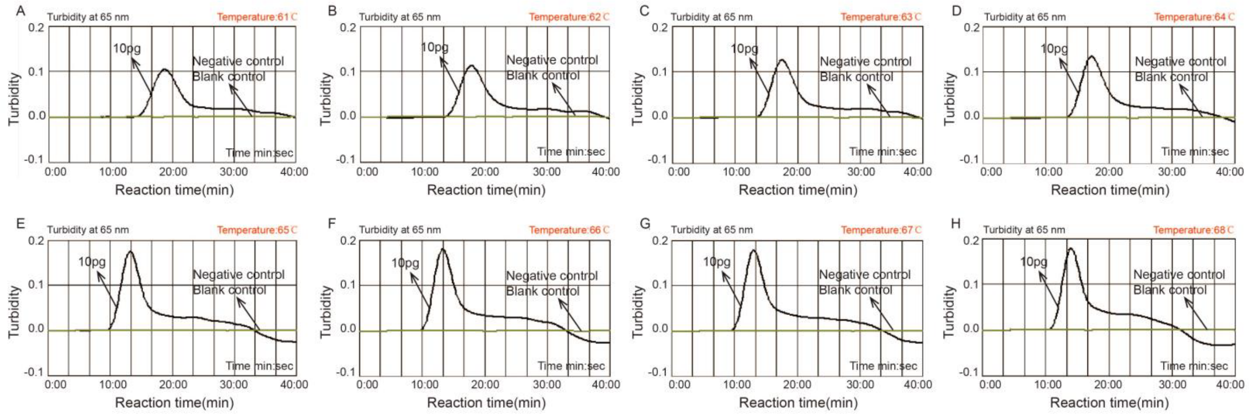

Supplementary Figures1s | Optimal amplification temperature for *H. influenzae*-LAMP assay. By using a real-time measurement to monitor the turbidity of *H. influenzae*-LAMP reactions. The corresponding curves were displayed in the panels. The negative control was *Candida albicans*, and the blank control was sterile double-distilled water. Abscissa represents reaction time (min), ordinate represents turbidity. The threshold value was 0.1, and the turbidity >0.1 was considered as positive amplification.Eight kinetic curves (A–H) were generated from 61 to 68◦C (1°C intervals), with 40 pg pUC57-Hi-OMP P6 DNA per reaction. The optimal *H. influenzae*-ERT-LAMP reaction temperature was 67 °C.
